# Remarkable Instance of Extra-Uterine Fœtation

**Published:** 1829-01-01

**Authors:** 


### CORK-STREET FEVER HOSPITAL.

### REMARKABLE INSTANCE OF EXTRA-UTERINE FŒTATION, WITH THE APPEARANCES ON DISSECTION.

*Case.* Ellen Bryan, aged 30, became pregnant in 1821, and about the expected period of parturition, felt something give way within her. The period passed, without delivery, and the size of the abdomen did not diminish. In a year and half afterwards, a second impregnation took place, and terminated in the birth of a full grown infant. In the course of two years more, Ellen Bryan gave premature birth to a live babe, of about six months intra-uterine existence. For the fourth time, she became pregnant, in 1826, and was delivered in the CORK-STREET HOSPITAL, after seven months utero-gestation. The original tumour remained unabated. She was re-admitted into the above institution, on the 19th of May, 1827, under Dr. O'Rearden. The following state of disease presented itself at this period.

Countenance pale, dejected, and expressive of long suffering; eyes sunken; much general emaciation; pulse slightly accelerated and extremely weak; abdomen of immense bulk and prominence, palpably enclosing a large solid substance like a fœtus; the centre of this prominency pointing forwards, and exhibiting an encrusted ulcer on its apex, a little below, and to the right side of the umbilicus. Diarrhœa. 21st. May — The encrustation at the apex of the above-mentioned prominency has detached itself and fallen off, and has left an aperture into the abdominal cavity from which there is a discharge of much fetid matter, partly ichorous and partly purulent. 27th. May.—Diarrhœa unabated: abdominal pain not acute, except when it is rendered so by pressure or by an unfavourable position of the patient; a most offensive odour emitted from the open abdominal ulcer—a gradually increasing dilatation of this aperture—a feeling, on the part of the patient, of being hurt interiorly by a bone. She takes a night pill containing one grain of opium; and, with a view of obviating as much as possible her hourly increasing debility, she is ordered four grains of sulphate of quinine twice a day, and twelve ounces of port wine, which she likes much. 9th. June—The abdominal aperture was hourly becoming larger. It was so wide yesterday as to expose to view the entire cause of this poor woman's tedious malady, viz: the inanimate body of a full grown male infant, which lay out of the womb in the abdominal cavity during more than the preceding six years. It has been taken out by a very intelligent surgeon, Mr. Trant, and is preserved in spirits of wine. It is covered with a semi-coriaceous cutis, which is particularly distinguished over almost the whole of the surface of the shoulders, back, and seat, by an adipocere-like appearance. Her pulse is scarcely perceptible. Portions of intestine have been destroyed by abrasion and sphacelation, and the fæces pass out anteriorly through the opening. 13th. June—Died.

*Dissection.*—Of the appearances exposed to view by the anatomical examination of the body, the following are the principal: An ample cyst, containing much pus, is situated behind and a little above the right side of the fundus uteri. The thick envelope of this sac consists in a prolongation of the peritoneum, especially of the right ligamentum latum. The right fallopian tube is distinctly traceable from both ends towards its centre, where it is connected with the external side of the parietes of the cyst, and is there lost in consequence of its being confounded with this envelope. The right ovarium is somewhat flattened on its posterior side, where it is close to the adjoining side of the cyst. The left ovarium, and corresponding fallopian tube are sound, and in their natural situations. The uterus is sound, and does not bear the slightest vestige of any former laceration, or of any kind of disease.

---

XLII.

